# Difference in long-term relapse rates between youths with ketamine use and those with stimulants use

**DOI:** 10.1186/s13011-018-0188-8

**Published:** 2018-12-22

**Authors:** Liang-Jen Wang, Mei-Yen Chen, Chin-Yin Lin, Mian-Yoon Chong, Wen-Jiun Chou, Yu-Han You, Chih-Pu Tsai, Yi-Syuan Chen, Shing-Fang Lu

**Affiliations:** 1 Department of Child and Adolescent Psychiatry, Kaohsiung Chang Gung Memorial Hospital and Chang Gung University College of Medicine, Kaohsiung, Taiwan; 2 Taiwan Kaohsiung Juvenile and Family Court, Kaohsiung, Taiwan; 3 Department of Psychiatry, Kaohsiung Chang Gung Memorial Hospital and Chang Gung University College of Medicine, Kaohsiung, Taiwan; 4 Department of Child and Adolescent Psychiatry, Kaohsiung Chang Gung Memorial Hospital, 123, Ta-Pei Road, Niao-Sung District, Kaohsiung City, 83301 Taiwan

**Keywords:** Substance abuse, Relapse, Adolescents, Ketamine, Stimulants, Longitudinal study

## Abstract

**Background:**

Understanding the relapse risk among different illicit drugs is vital for developing an adequate relapse prevention policy. Therefore, the current study aims to explore the potential difference in long-term relapse rates between youths who use ketamine and those who use stimulants (3,4-Methylenedioxymethamphetamine [MDMA] or methamphetamine).

**Methods:**

The study’s participants included 92 youths with ketamine use (ketamine group, mean age: 16.0 years) and 43 youths with MDMA/methamphetamine use (stimulants group, mean age: 16.1 years) that had undergone a family-oriented treatment program in a medical center in Taiwan. All participants were followed up for a maximum of 7 years in order to observe their long-term outcomes with regard to substance use relapse.

**Results:**

During the follow-up period, compared to the 34.8% relapse rate in ketamine users, their counterparts who used MDMA or methamphetamine had a significantly higher relapse rate (60.5%, Adjusted HR = 1.86, 95%CI: 1.06–3.28, *p* = 0.032). Of the youths in the ketamine group that relapsed, 65.6% continued to use ketamine in their relapse event, while 34.4% switched to MDMA or methamphetamine. Among the relapsing youths in the stimulants group, 84.6% continued to use MDMA or methamphetamine in their relapse event, while 15.4% switched to ketamine (*p* = 0.042).

**Conclusions:**

Compared to adolescents who use ketamine, those using MDMA or methamphetamine had higher relapse rates and were more likely to use the same type of drug upon relapsing. These results can serve as a crucial reference for developing relapse prevention policies of illicit drugs for the youth population.

**Electronic supplementary material:**

The online version of this article (10.1186/s13011-018-0188-8) contains supplementary material, which is available to authorized users.

## Background

Youths’ abuse of various substances has emerged as a worldwide public health issue [1]. In the United States alone, as many as 75.6% of youths under the age of 18 have admitted to using an addictive substance (such as nicotine in cigarettes, alcohol, marijuana, and/or cocaine) at least once [2, 3]. Of the various illicit drugs available, marijuana remains the most widely consumed in the United States [4]. In East Asia, ketamine, 3,4-methylenedioxymethamphetamine (MDMA), and methamphetamine have become some of the most popular recreational drugs [5–8]. In Taiwan, ketamine and MDMA have emerged as the most commonly used illegal drugs among youths [9, 10]. Compared to certain hard drugs, such as heroin or cocaine, these club drugs are relatively cheap and easy to obtain. As a result, youths already at a high risk of substance abuse are very likely to use these drugs [11]. Substance abuse can result in serious and harmful complications in youths throughout their entire lives, including physical illnesses, cognitive impairments, and issues with academic and occupational function, resulting in social burden and, in some cases, even death [12, 13]. Therefore, understanding the risk of relapse among various illicit drugs is essential for developing adequate policies for preventing substance reuse.

Ketamine, an anesthetic and analgesic with hallucinogenic effects developed in the 1960s, has recently become a very popular recreational drug among adolescents [11, 14]. A noncompetitive antagonist of N-methyl-D-aspartate receptor, ketamine has been clinically shown to have a significant and rapid antidepressant effect [15]. However, chronic ketamine abuse has both physical and psychological risks, including ulcerative cystitis, kidney and gastrointestinal dysfunction, cognitive impairments, psychosis, depression, and psychological cravings [16, 17]. Therefore, ending ketamine abuse and preventing relapse is vital to reducing related illnesses in adolescents, as well as the burden they may place on society [18]. However, no study has yet reported relapse rates among adolescents using ketamine for recreational purposes.

Methamphetamine and MDMA have similar chemical structures and pharmacologic properties. Both are psychostimulants used primarily for recreation [19]. The recreational effects of MDMA, also known as ecstasy, include increased empathy, euphoria, and heightened sensations. Methamphetamine is commonly abused for its stimulant, euphoric, empathogenic, and hallucinogenic properties. The majority of these effects are caused by acute increases in dopamine and serotonin neurotransmission. As a result, both methamphetamine and MDMA permanently damage dopamine and serotonin nerve terminals [20]. Chronic use of methamphetamines can have such serious health consequences as cognitive impairment, poor health status, increased social burden, and a higher risk of mortality [21, 22]. Furthermore, methamphetamine and MDMA are both highly addictive, and patients treated for methamphetamine and MDMA dependence often relapse [23–25].

Little is known about whether the adverse effects of ketamine and stimulants (methamphetamine/MDMA) on recreational users differ. Some researchers have developed and explored the feasibility of assessing the harm caused by various illicit drugs in an evidence-based fashion. Their drug ranking revealed that ketamine may be more harmful than amphetamine [26, 27]. However, according to the Controlled Drugs Act in Taiwan, ketamine is a schedule 3 illicit drug, while MDMA/methamphetamine are both schedule 2 illicit drugs, which indicates that MDMA/methamphetamine are considered more toxic, addictive, or harmful than ketamine in Taiwan’s regulatory system. However, evidence that compares the relapse rate of ketamine users with that of methamphetamine or MDMA users in the real world is still lacking.

Youths with substance abuse issues are generally considered a form of juvenile delinquency and may be sentenced to probation, to receive reformatory education, or to undergo detoxification. To combat this problem, Taiwan Kaohsiung Juvenile and Family Court and Kaohsiung Chang Gung Memorial Hospital have been working together since 2010 on a specific treatment program that targets both the substance-using juveniles and their caregivers. The court act requires that underage individuals arrested for substance abuse undergo a treatment program in a hospital. In this study, the respective program consists of 10 weekly psychotherapy sessions based on motivational enhancement principles [28], as well as 10 weekly parental skill training sessions [29, 30] for the youths’ caregivers. The details of this family-oriented treatment program and its effectiveness on youths with substance abuse issues and their caregivers have been provided in previous studies [31, 32]. The adolescents who have completed the family-orientated treatment program also received long-term follow-up in the justice system, which offers us a great opportunity to determine the relapse risk between ketamine users and those who use stimulants (methamphetamine or MDMA). Therefore, the purpose of the current study is to investigate the potential difference in long-term relapse rates (up to 7 years) between ketamine-using and stimulants-using youths.

## Methods

### Study participants

The participants in this research are adolescents with substance use who appeared before Taiwan’s Kaohsiung Juvenile and Family Court and were sentenced to undergo a weekly 10-session out-patient family-oriented treatment program at Kaohsiung Chang Gung Memorial Hospital from 2010 to 2016. Judges order underage individuals arrested for substance use to participate in a hospital-run treatment program. The inclusion criteria were as follows: (1) aged between 12 and 18 years old; (2) having illicit drug use; and (3) the youths and at least one of their caregivers could attend the treatment program. The exclusion criteria included (1) intellectual disability, (2) having apparent psychotic symptoms, and (3) having first-class illicit drug use (i.e., heroin, morphine, or cocaine). Upon completing the treatment program, the youths received supervision by the protection officers of Taiwan’s Kaohsiung Juvenile and Family Court, who provided them with moral education and counseling in their work or studies afterwards.

### The family-orientated treatment program for adolescents

The participating youths were required to attend one session a week for 10 weeks of a group program for relapse prevention based on motivational enhancement (MET) concepts and led by two experienced hospital psychologists. Each session had approximately eight participants and lasted for 120 min. The goal of these sessions was to awaken the youths’ motivation for change by discovering not only their reasons for using but also reasons for abstaining. The principal method for each session was feedback, with reflection and questions used to prompt self-motivational statements.

The parental skill training (PST) program for the sentenced youths’ caregivers also consisted of 10 weekly, 120-min sessions that were led by two senior consulting psychologists assigned by the court. These therapists helped the caregivers to assess their current relationship with their children and their techniques with regard to influencing them. Furthermore, these therapists supported the participating caregivers in discovering their negative family interaction patterns so that they could change them and thus their family’s daily environment. The therapists also shared new methods with the caregivers for reaching out to their teenaged children and techniques for helping their children address the issues that divide them in developmentally non-normative ways from their caregivers.

### Study procedures and outcomes

The participating youths’ socio-demographic characteristics (e.g., categories of substances being used, history of previous convictions, family status, and academic or social status) were provided by Taiwan’s Kaohsiung Juvenile and Family Court. Upon completing the course of treatment, all participants were submitted to the supervision and probation of the court, which consisted of notifying the protection officers of their academic, social, and living status approximately once a month. During the follow-up period, the adolescents were required to provide urine samples to be tested for the presence of the substance at the discretion of the judges or protection officers. If the aforementioned urine test came back positive, the court would give the adolescent a hearing and then either sentence him or her to participate in reformatory education or be incarcerated in the detoxification unit of the detention center. Relapse was defined with a positive urine test, and relapse events were recorded in a nationwide judicial electronic system. We followed participants’ records through December 31, 2017. The outcome of this study was concerned with substance–use relapse during the follow-up period.

### Statistical analysis

The data in this study were analyzed using the statistical software package SPSS, version 16.0 (SPSS Inc., Chicago, IL, USA). Variables are presented as either mean ± standard deviation (SD) or frequency. A chi-square (χ^2^) test or independent t-test was used to compare variables between youths using ketamine (ketamine group) and those using methamphetamine or MDMA (stimulants group). We adopted the McNemar-Bowker Test to investigate the proportion of switching substance use among individuals with substance–use relapse. Subjects were categorized into those who used the same substance as their index substance use and those who switched substances.

Each youth’s index episode of substance use within the study period was used to calculate risk over time. As for survival analysis, the time function was defined as the number of days from the index substance use to the end of the period for those youths who had no other instance of substance use to that point or to the date of relapse if before the end of the follow-up period. During the follow-up period, cumulative survival rates were expressed using Kaplan-Meier curves. We developed a Cox regression model to estimate the treatment effects on relapse controlling for socio-demographic variables. Adjusted hazard ratios (aHR) were calculated with 95% confidence intervals (CI). We considered a two-tailed *p*-value less than 0.05 statistically significant.

## Results

Of the 135 youths that underwent the family-oriented treatment, 92 of them used ketamine in their index event and were thus categorized into the ketamine group. The remaining 43 youths used MDMA or methamphetamine and were labeled as the stimulants group. Table 1 provides the socio-demographic characteristics between the ketamine group (mean age: 16.0 years, 77.2% were boys) and the stimulants group (mean age: 16.1 years, 76.7% were boys) at baseline. Regarding academic or social status, 39.1 and 16.3% were attending school; 30.4 and 51.2% were employed; and 30.4 and 32.6% had been suspended or had already dropped out from school among the ketamine group and stimulants group, respectively.

**Table 1:** Baseline socio-demographic characteristics of the adolescents who used ketamine (ketamine group) and those who used methamphetamine or MDMA (stimulants group)

	Ketamine group (*N* = 92)	Stimulants group (*N* = 43)	Statistic	*P*-value
Age (years)				
Range	13–17	12–17		
Mean (SD)	16.0 (1.0)	16.1 (1.1)	*t* = 0.669	0.505
Gender			χ^2^ = 0.003	0.956
Female	21 (22.8)	10 (23.3)		
Male	71 (77.2)	23 (76.7)		
Previous conviction record at baseline			χ^2^ = 0.181	0.670
Without	63 (68.5)	31 (72.1)		
With	29 (31.5)	12 (27.9)		
Academic or social status at baseline			χ^2^ = 8246	0.016*
Attending school	36 (39.1)	7 (16.3)		
Employed	28 (30.4)	22 (51.2)		
Dropout and unemployed	28 (30.4)	14 (32.6)		
Family status			χ^2^ = 0.545	0.761
Double-parent families	42 (45.7)	19 (44.2)		
Single-parent families	38 (41.3)	20 (46.5)		
Grandparent(s)	12 (13.0)	4 (9.3)		

*SD* standard deviation, *MDMA* 3,4-methylenedioxy-methamphetamine

Of the 135 participants, 58 (43%) of them had a substance abuse relapse during the follow-up period (Table 2). The relapse rate in the stimulants group (60.5%) was significantly greater than that in the ketamine group (34.8%) (*p* = 0.005). Of the 32 youths in the ketamine group who relapsed, 21 (65.6%) continued using ketamine in their relapse event, while 11 (34.4%) switched to MDMA or methamphetamine. Of the 26 youths in the stimulants group who relapsed, 22 (84.6%) continued using MDMA or methamphetamine in their relapse event, while four (15.4%) switched to ketamine.

**Table 2:** Relapse in substance use of adolescents after their index substance use during the follow-up period

Relapse after the index substance use	Ketamine group (*N* = 92)	Stimulants group (*N* = 43)	Statistic ^a^	*P*-value
No	60 (65.2)	17 (39.5)	7.888	0.005*
Yes	32 (34.8)	26 (60.5)		
Substance use in the relapse event	Ketamine group (*N* = 32)	Stimulants group (*N* = 26)	Statistic ^b^	*P*-value
Ketamine	21 (65.6)	4 (15.4)	14.764	0.042*
Stimulants (MDMA or methamphetamine)	11 (34.4)	22 (84.6)		

^a^Chi-square (χ^2^) test; ^b^ McNemar-Bowker Test; *p < 0.05

**Fig. 1:**
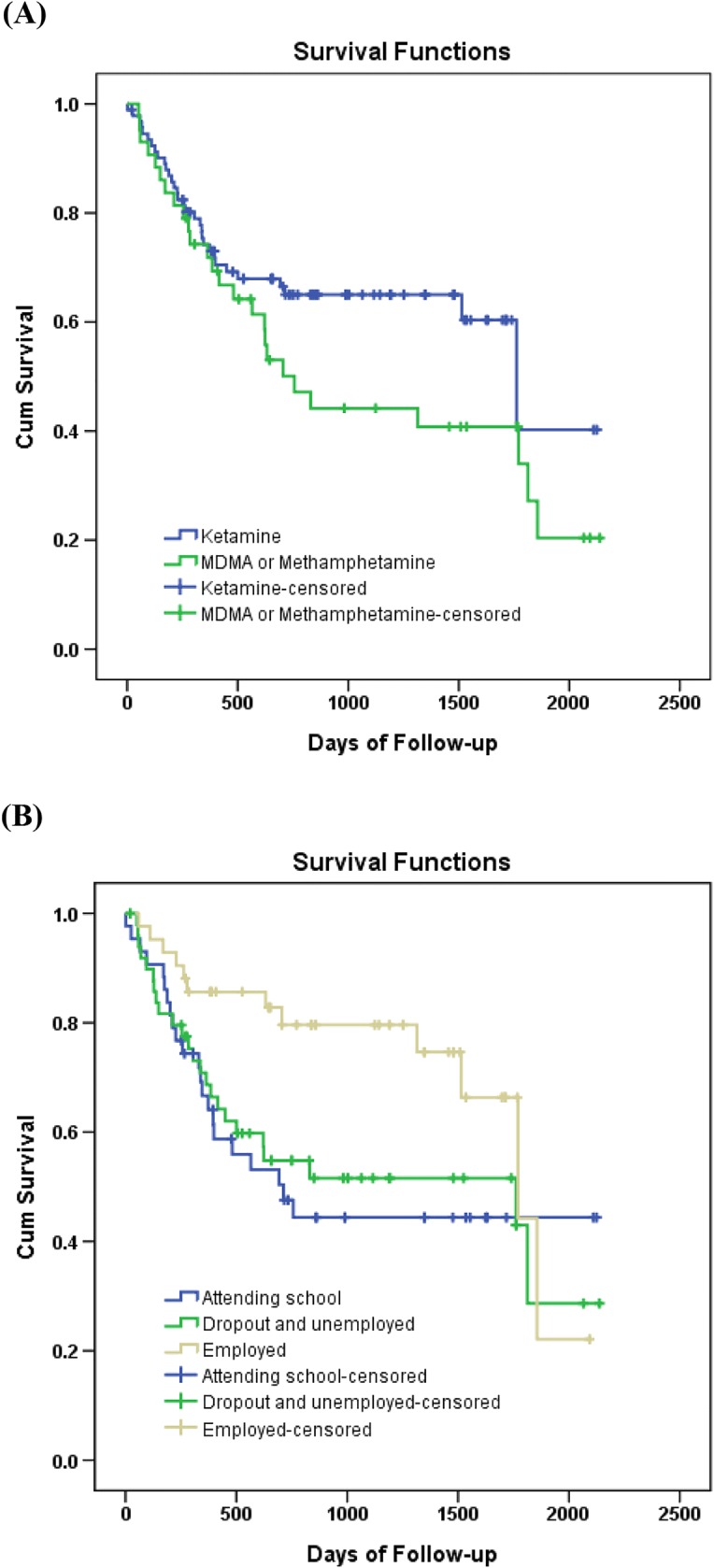
Kaplan-Meier curves of substance use relapse during the follow-up period categorized into ketamine users and stimulants users (**a**) and into academic or social status at the baseline (**b**)

The Cox proportional hazard models of the risk of relapse in the follow-up period are shown in Table 3. Compared to the youths that used ketamine at the index event, their counterparts who used MDMA or methamphetamine had a significantly higher risk of relapse (aHR = 1.86, 95%CI: 1.06–3.28, *p* = 0.032). Furthermore, the youths’ academic or social status at baseline also significantly predicted the rates of relapse. Compared to the subjects employed at baseline, youths who were attending school at the baseline were more likely to relapse in substance abuse during the study period (aHR = 2.77, 95%CI: 1.28–6.02, *p* = 0.010). Figure 1 shows the Kaplan-Meier curves of relapse during the follow-up period categorized by substance use at the index event (Fig. 1a) and by academic or social status at baseline (Fig. 1b).

**Table 3:** Risk of relapse after the index substance use for related variables estimated by Cox proportional hazards model

Variables	Relapse	Unadjusted model		Adjusted model	
	n/N (%)	HR (95% CI)	*P*-value	aHR (95% CI)	*P*-value
Age (years)	–	0.91 (0.72–1.16)	0.450	1.00 (0.77–1.28)	0.968
Gender					
Male	47/104 (45.2)	1		1	
Female	11/31 (35.5)	0.83 (0.43–1.60)	0.577	0.89 (0.45–1.77)	0.735
Substance use					
Ketamine	32/92 (34.8)	1		1	
Stimulants	26/43 (60.5)	1.58 (0.93–2.68)	0.090	1.86 (1.06–3.28)	0.032*
Previous conviction record					
Without	36/94 (38.3)	1		1	
With	22/41 (53.7)	1.48 (0.87–2.51)	0.152	1.56 (0.91–2.67)	0.106
Academic or social status					
Employed	12/42 (28.6)	1		1	
Attending school	22/43 (51.2)	2.23 (1.10–4.52)	0.025*	2.77 (1.28–6.02)	0.010*
Dropout and unemployed	24/50 (48.0)	2.06 (1.03–4.12)	0.042*	1.99 (0.97–4.08)	0.061
Family status					
Double-parent families	26/61 (42.6)	1		1	
Single-parent families	26/58 (44.8)	1.15 (0.67–1.97)	0.624	1.16 (0.66–2.02)	0.608
Grandparent(s)	6/16 (37.5)	0.81 (0.33–1.97)	0.637	0.72 (0.28–1.86)	0.492

Stimulants: methamphetamine or MDMA; HR: unadjusted hazard ratio; aHR: adjusted hazard ratio; 95% CI, 95% confidence interval; *n*, number of individuals who relapsed with substance use; *N*, number of total subjects; *p < 0.05

## Discussion

### Substance use relapse

To the best of our knowledge, this study is among the first to compare the long-term relapse rates of adolescents with ketamine use and those with stimulants use. Of all the participants, 43% relapsed during the study period (up to 7 years of follow-up). Substance use relapse was defined as subsequent conviction due to a positive urine test result. We believe the outcome measure in this study to be objective and reliable. For youths with cannabis use, the recurrence rate was relatively modest (27.7%) and was most likely to occur within the first 36 months after the first usage episode [33]. Meanwhile, a cohort study conducted in southern Brazil revealed that adolescents with crack cocaine abuse or dependence had alarmingly high relapse rates in both the first (65.9%) and third months (86.4%) [34]. Opioids are highly addictive substances from which abstaining is extremely difficult; thus, they can have devastating consequences for young people and their families [35]. Taking together with our findings, although the substance-using adolescents received family-oriented intervention plus court supervision, substance abuse relapses are still common in the real world. Therefore, breaking the drug addiction cycle for substance-using adolescents remains a challenge [36].

We found that the ketamine group had a lower relapse rate (34.8%) than the stimulants group (60.5%), which can be explained by a number of factors. First, the difference in relapse risk was associated with the pharmacological properties of ketamine and stimulants. The intensity of the withdrawal symptoms and euphoric effects derived from ketamine may be less than methamphetamine or MDMA, so the users’ craving behaviors toward ketamine may also be less, thus leading to a lower relapse rate. Second, ketamine use likely precedes the use of other illicit substances and the development of addiction to other such substances [37]. Furthermore, adolescents more vulnerable to taking drugs may simply be more likely to start with readily available substances like ketamine, while their subsequent social interactions with others who use drugs may increase their opportunities to try other drugs [11]. Therefore, relative to methamphetamine/MDMA users, ketamine users may have been in the earlier stage of substance use and may have had greater potential to achieve abstinence from drug abuse [38]. Third, the treatment program in our study combined MEP and PST programs to enhance participants’ family function and further prevent relapse of substance use. Ketamine users most likely had a better response to this psychosocial intervention, and so their risk of substance use relapse was reduced more than that of stimulants users.

Of the youths who relapsed in their substance use, those in the stimulants group were more likely to use the same class of substance in their relapse event (84.6%) compared to the ketamine group (65.6%). In other words, ketamine users were more likely to switch the substance used in their relapse event (34.4%) when compared to the stimulants group (15.4%). This finding suggests that ketamine users may experiment with methamphetamines or MDMA in a subsequent drug use event. However, once youths have tried methamphetamines or MDMA, they are less likely to go back to the previously used soft drugs, and the possibility of drug abstinence thus decreased. The findings in this study partially support the policy of classifying ketamine and stimulants into different levels of illicit drugs.

### Academic or social status

Adolescents who were already employed had the lowest relapse rates (28.6%), followed by those who had dropped out of school and were unemployed (48%); youths who were attending school at the baseline had the highest relapse rate (51.2%). Adolescents with illicit drug use are at a significant risk of dropping out of school, social maladjustment, and occupational impairments [39, 40]. Previous studies have indicated that unemployment is a risk factor for substance use disorders [41]; in contrast, substance use may also have a detrimental impact on occupational function among young adults [42]. Our study’s findings reveal that employment was a protective factor for substance use relapse among the adolescent population, indicating that youths who were employed, relative to those attending school, may have better lifestyles and greater motivation to cease substance use.

We found that the youths attending school at the baseline had the highest relapse rate (51.2%), even higher than those who had dropped out and were unemployed (48%). This particular finding is unexpected and goes against our empirical imagination. Youths who recover from substance use mostly improve or change their lifestyles, which is achieved by making better behavioral choices and exerting personal control over their behavior [43]. We also observed that having peers that used illicit drugs was a risk factor that contributed to substance use [14, 44]. We supposed that the youths who attended school were more likely to experience pressure from peers using illicit drugs and had easy access to recreational drugs through their classmates [45]. These underlying factors may be associated with the increased relapse rates in our study population.

### Limitations and strengths

This study has several limitations that should be mentioned at this point. First, this study had a small sample size. The reduced statistical power restricts its ability to identify outcome-associated factors. Second, ketamine use and stimulants use were not allocated through randomization. The demographic characteristics and sample sizes differed between the two groups at baseline. The results could be unwittingly influenced by selection bias. However, due to ethical research concerns, a human study that investigates this topic is impossible to administer using a randomized-controlled method. Third, we compared the relapse rates between the ketamine group and the stimulants group using long-term follow-up data after receiving family treatment under court supervision. The relapse situation among substance-using youths in the community who did not attend the treatment program remains unclear. Fourth, due to the small sample size, we categorized methamphetamine users and MDMA users into a single group. However, upon performing sensitivity tests (Additional file 1: Table S1), we discovered that the relapse rates of both methamphetamine users (56.8%) and MDMA users (83.3%) were still higher than that of ketamine users (34.8%). This discovery indicates that our findings regarding relapse rates were not confounded by stimulant category. Finally, several additional factors that may have potentially influenced the severity of the drug addiction and the study’s outcomes were not identified, such as peer relationships [14], intelligence or academic performance [45], and psychiatric comorbidities (attention-deficit/hyperactivity disorder or depression) [46]. Therefore, we were unable to determine whether these factors affected the results of this study.

## Conclusion

Despite its limitations, this study is the foremost in providing evidence about the long-term relapse rates among youths with recreational use of ketamine and stimulants (MDMA and methamphetamine). This study shows that adolescents with MDMA or methamphetamine use had higher relapse rates when compared to adolescents who use ketamine during as many as 7 years of follow-up and tended to use the same type of drugs at their relapse events. Our findings in this study support classifying ketamine and stimulants into different illicit drug categories. This study can be used as a reference for developing relapse prevention policies for illicit drug use among adolescents.

## Additional file

Additional file 1: **Table S1.** Relapse in substance use of adolescents after their index substance use during the follow-up period. (DOC 48 kb)

## Abbreviations

MDMA: 3,4-Methylenedioxymethamphetamine
MET: motivational enhancement
PST: parental skill training
