# Supplementary material for: Difference in long-term relapse rates between youths with ketamine use and those with stimulants use
Source: Subst Abuse Treat Prev Policy. 2018 Dec 22;13:50. doi: 10.1186/s13011-018-0188-8 (PMC6303878; doi:10.1186/s13011-018-0188-8)
Supplement: Supplementary file 1 — Table S1. Relapse in substance use of adolescents after their index substance use during the follow-up period. (DOC 48 kb) [file 13011_2018_188_MOESM1_ESM.doc]

**Additional file 1: Table S1.** Relapse in substance use of adolescents after their index substance use during the follow-up period

| **Relapse after the index substance use** | **Ketamine group (N=92)** | **MDMA group (N=6)** | **Methamphetamine group (N=37)** | **Statistic ^a^** | ***P*-value** |
| --- | --- | --- | --- | --- | --- |
| No | 60 (65.2) | 1 (16.7) | 16 (43.2) | 9.105 | 0.008* |
| Yes | 32 (34.8) | 5 (83.3) | 21 (56.8) |
| **Substance use in the relapse event** | **Ketamine group (N=32)** | **MDMA group (N=5)** | **Methamphetamine group (N=21)** | **Statistic ^a^** | ***P*-value** |
| Ketamine | 21 (65.6) | 1 (20) | 3 (14.3) | 14.917 | <0.001* |
| Stimulants | 11 (34.4) | 4 (80) | 18 (85.7) |

Stimulants: MDMA or methamphetamine; ^a^ Chi-square (χ^2^) test; *p<0.05
